# Fluorescent markers of the endocytic pathway in *Zymoseptoria tritici*^☆^

**DOI:** 10.1016/j.fgb.2015.03.019

**Published:** 2015-06

**Authors:** S. Kilaru, M. Schuster, M. Latz, M. Guo, G. Steinberg

**Affiliations:** Biosciences, University of Exeter, Exeter EX4 4QD, UK

**Keywords:** Tub2, α-tubulin, Enhanced green-fluorescent protein, eGFP, Zt, *Zymoseptoria tritici*, *sdi1*, succinate dehydrogenase 1, RB and LB, right and left border, mCherry, monomeric cherry, *hph*, hygromycin phosphotransferase, *nptII*, neomycin phosphotransferase, *bar*, phosphinothricin acetyltransferase, Endosomes, Actin patches, Pathogenic fungi, Septoria tritici blotch, *Mycosphaerella graminicola*

## Abstract

Hyphal growth in filamentous fungi is supported by the uptake (endocytosis) and recycling of membranes and associated proteins at the growing tip. An increasing body of published evidence in various fungi demonstrates that this process is of essential importance for fungal growth and pathogenicity. Here, we introduce fluorescent markers to visualize the endocytic pathway in the wheat pathogen *Zymoseptoria tritici*. We fused enhanced green-fluorescent protein (eGFP) to the actin-binding protein fimbrin (ZtFim1), which is located in actin patches that are formed at the plasma membrane and are participating in endocytic uptake at the cell surface. In addition, we tagged early endosomes by eGFP-labelling a Rab5-homologue (ZtRab5) and late endosomes and vacuoles by expressing eGFP-Rab7 homologue (ZtRab7). Using fluorescent dyes and live cell imaging we confirmed the dynamic behavior and localization of these markers. This set of molecular tools enables an in-depth phenotypic analysis of *Z. tritici* mutant strains thereby supporting new strategies towards the goal of controlling wheat against *Z. tritici*.

## Highlights

• We establish *Z. tritici* fimbrin (ZtFim1) and small GTPases (ZtRab5, ZtRab7) as endocytic markers.
• All markers localize correctly, proven by live cell imaging and co-staining and pharmaceutical studies.
• We provide 3 carboxin-resistance conveying vectors for integration of all markers into the *sdi1* locus.
• We provide 3 hygromycin B-resistance conveying vectors for random integration of all markers.

## 1 Introduction

Tip growth is a hallmark of filamentous fungi, used to explore soil, exploit substrates or invade host organisms during fungal pathogenesis (Steinberg, 2007). Tip growth is characterized by polar extension of the hyphal growth region, which requires constant delivery of growth supplies, such as membranes and proteins, but also protein complexes and cell wall-forming enzymes. It is widely accepted that tip growth involves the delivery of Golgi-derived secretory vesicles, which are transported to the hyphal tip and accumulate in the Spitzenkörper (Harris et al., 2005; Riquelme and Sanchez-Leon, 2014). This process involves cytoskeleton and molecular motors, which utilize ATP to transport the membranous cargo to the tip for polarized secretion (Egan et al., 2012a; Steinberg, 2007; Xiang and Plamann, 2003). The discovery of endocytosis in the late 1990s added another important process to the mechanism of tip growth. It was shown firstly in *Ustilago maydis* that early endosomes participate in fungal morphogenesis and hyphal tip growth (Wedlich-Söldner et al., 2000). Subsequent studies showed that these endosomes participate in apical recycling of receptors at the hyphal tip (Fuchs et al., 2006). Shortly thereafter, evidence for apical endocytic recycling in fungal growth and morphology was found in filamentous ascomycetes (Higuchi et al., 2009; Lee et al., 2008; Araujo-Bazán et al., 2008; Upadhyay and Shaw, 2008), which led to the concept of an apical recycling model (Shaw et al., 2011; overview in Penalva, 2010; Steinberg, 2014). Interestingly, early endosomes move bi-directionally along microtubules (Wedlich-Söldner et al., 2000), a process driven by kinesin-3 and dynein (Wedlich-Söldner et al., 2002; Lenz et al., 2006; Abenza et al., 2009; Zekert and Fischer, 2009; Zhang et al., 2010; Egan et al., 2012b; overview in Steinberg, 2014). Recent work in the corn smut fungus *U. maydis* has shed light on the function of this motility. Surprisingly, it demonstrates that this motility distributes the protein translation machinery, including mRNA (Baumann et al., 2012) and ribosomes (Higuchi et al., 2014), which is required for extended hyphal growth. In addition, long-range motility of early endosomes mediates communication between the invading hyphal tip and the nucleus (Bielska et al., 2014). This long-range signaling is required for production of effector proteins and, therefore, is essential for virulence of *U. maydis* (overview in Higuchi and Steinberg, 2015).

**Fig. 1:**
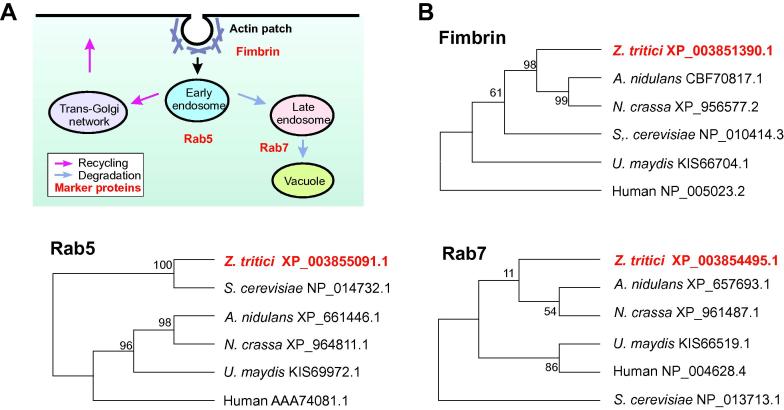
Markers for the endocytic pathway in *Z. tritici*. (A) Schematic overview of the main endocytic membrane trafficking pathways in eukaryotes. Marker proteins for endocytic organelles are indicated in red. Note that the diagram is a highly simplified. (B) Phylogenetic trees comparing the predicted full-length amino acid sequence of homologues of the actin-binding protein fimbrin, and the endocytic small GTPases Rab5 and Rab7 in fungi and humans. The *Z. tritici* orthologues, used in this study, are indicated in bold and red. NCBI accession numbers are given (http://www.ncbi.nlm.nih.gov/pubmed). Maximum likelihood trees were generated using MEGA5.1 (Tamura et al., 2011). Bootstrap values are indicated at branching points.

Early endosomes are part of the endocytic pathway. This begins with the uptake of membranes and fluid at the plasma membrane (Fig. 1A). Endocytosis in yeasts and filamentous fungi involve polar-localized actin patches (Warren et al., 2002; Araujo-Bazán et al., 2008; Basu et al., 2014). The actin-binding protein fimbrin localizes to these actin patches (Wu et al., 2001; Castillo-Lluva et al., 2007; Delgado-Alvarez et al., 2010; Upadhyay and Shaw, 2008) and performs essential roles in the formation of endocytic vesicles at the plasma membrane (Shaw et al., 2011; Skau and Kovar, 2010). Endocytic vesicles deliver their cargo to early endosomes, which in animals and fungi carry the small GTPase Rab5 (Fig. 1A; Fuchs et al., 2006; Abenza et al., 2009; Chavrier et al., 1990; Seidel et al., 2013; Zerial and McBride, 2001). Rab5-positive early endosomes mature to late endosomes, which in animals and fungi carry the small GTPase Rab7 (Abenza et al., 2012; Chavrier et al., 1990; Higuchi et al., 2014). This compartment is an intermediate before endocytosed material is delivered to the vacuole for degradation.

In this study, we introduce fluorescent marker proteins for visualization of the endocytic pathway in the ascomycete *Zymoseptoria tritici*. This fungus is a major pathogen on wheat, causing significant economic damage in the European Union (Gurr and Fones, 2015) and, consequently, is considered amongst the most devastating plant pathogenic fungi (Dean et al., 2012). We confirm the specific localization of all markers using dual-color live cell imaging, pharmacological experiments and *in vivo* analysis of their cellular dynamics. We also describe 6 vectors, carrying 2 different resistance cassettes, to enable phenotypic analyses of morphological *Z. tritici* mutants or in-depth mode of action studies on novel anti-fungal chemistries.

## 2 Materials and methods

### 2.1 Bacterial and fungal strains and growth conditions

*Escherichia coli* strain DH5α was used for the maintenance of plasmids. *Agrobacterium tumefaciens* strain EHA105 (Hood et al., 1993) was used for maintenance of plasmids and subsequently for *A. tumefaciens*-mediated transformation of *Z. tritici*. *E. coli* and *A. tumefaciens* were grown in DYT media (tryptone, 16 g/l; yeast extract, 10 g/l; NaCl, 5 g/l; with 20 g/l agar added for preparing the plates) at 37 °C and 28 °C respectively. The fully sequenced *Z. tritici* wild-type isolate IPO323 (Goodwin et al., 2011) was used as recipient strain for the genetic transformation experiments. The isolate was inoculated from stocks stored in glycerol (NSY glycerol; nutrient broth, 8 g/l; yeast extract, 1 g/l; sucrose, 5 g/l; glycerol, 700 ml/l) at −80 °C onto solid YPD agar (yeast extract, 10 g/l; peptone, 20 g/l; glucose, 20 g/l; agar, 20 g/l) and grown at 18 °C for 4–5 days.

### 2.2 Identification of *Z. tritici* homologues and bioinformatics

To identify homologues of the chosen marker proteins, we screened the published sequence of *Z. tritici* strain IPO323 (http://genome.jgi.doe.gov/Mycgr3/Mycgr3.home.html), using the provided BLASP function and the *U. maydis* proteins sequences of Fim1 (NCBI accession number: XP_760915.1), Rab5a (NCBI accession number: XP_757052.1) and Rab7 (NCBI accession number: 761658.1). Sequences were obtained from the NCBI server (http://www.ncbi.nlm.nih.gov/pubmed) and comparison was done using CLUSTAL W (http://www.ebi.ac.uk/Tools/msa/clustalw2/) and EMBOSS Needle (http://www.ebi.ac.uk/Tools/psa/emboss_needle/) and domain structures were analyzed in PFAM (http://pfam.xfam.org/search/sequence). Finally, phylogenetic trees were generated in MEGA5.2, using a Maximum likelihood algorithm, followed by 1000 bootstrap cycles (http://www.megasoftware.net/; (Tamura et al., 2011).

### 2.3 Molecular cloning

All the vectors used in this study were generated by *in vivo* recombination in the yeast *Saccharomyces cerevisiae* DS94 (MATα, *ura3-52*, *trp1-1*, *leu2-3*, *his3-111*, and *lys2-801* (Tang et al., 1996) following published procedures (Raymond et al., 1999). PCR reactions and other molecular techniques followed standard protocols (Sambrook and Russell, 2001). All restriction enzymes and reagents were obtained from New England Biolabs Inc (NEB, Herts, UK).

**Fig. 2:**
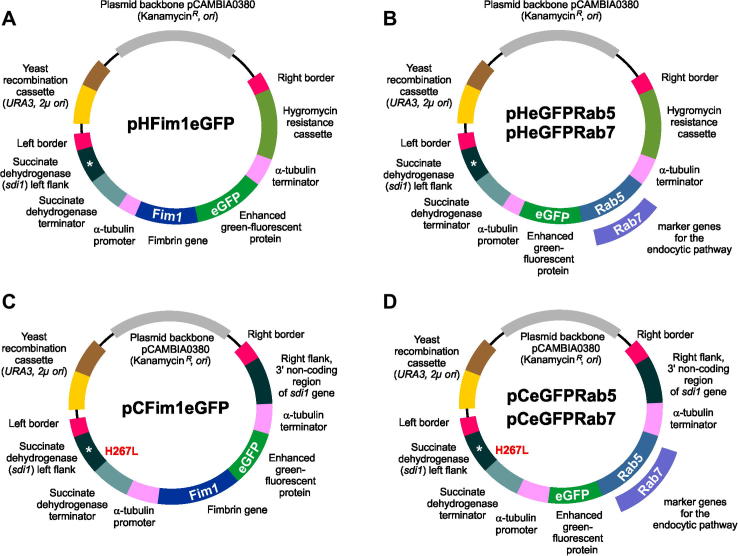
Vectors to investigate the endocytic pathway in *Z. tritici*. (A and B) Vectors for random ectopic integration of endocytic marker constructs into the genome of *Z. tritici*. The vectors pHFim1eGFP, pHeGFPRab5 and pHeGFPRab7 confer hygromycin resistance and are designed for random ectopic integration of GFP-marker fusion proteins into the genome of *Z. tritici*. Note that these vectors were derived from carboxin resistance conferring vectors. As such they contain part of the succinate dehydrogenase gene, carrying the mutation H267L and succinate dehydrogenase terminator. However, these fragments are of no significance. (C and D) Vectors for targeted integration of endocytic marker constructs into the *sdi1* locus of *Z. tritici*. The vectors pCFim1eGFP, pCeGFPRab5 and pCeGFPRab7 contain the H267L point mutation in a stretch of *sdi1* sequence, which confers carboxin resistance and allows targeted integration into the *sdi1* locus of *Z. tritici* (for more information see Kilaru et al., 2015). Note that fragments are not drawn to scale. For more accurate information on fragment sizes see main text.

Vector pHFim1eGFP contains *egfp* fused to the full-length *ztfim1* under the control of constitutive *zttub2* promoter and terminator sequences for random ectopic integration into the genome of *Z. tritici* using hygromycin B as selection agent. A 13,159 bp fragment of pHeGFPTub2 (see Schuster et al., 2015; digested with *Bsr*GI), 1149 bp *zttub2* promoter (amplified with SK-Sep-14 and SK-Sep-47; Table 2) 2073 bp full length *ztfim1* gene without stop codon (amplified with SK-Sep-240 and SK-Sep-241; Table 2) and 720 bp *egfp* (amplified with SK-Sep-16 and SK-Sep-42; Table 2) were recombined in yeast *S. cerevisiae* to obtain the vector pHFim1eGFP (Fig. 2A) Note that this vector was derived pHeGFPTub2, which a derivative of carboxin resistance conferring vector and as such it contain part of the succinate dehydrogenase gene, carrying the mutation H267L and succinate dehydrogenase terminator. However, these fragments are of no significance.

The vector pCFim1eGFP contains *egfp* fused to the full-length *ztfim1* under the control of constitutive *zttub2* promoter and terminator sequences for targeted integration into the *sdi1* locus of *Z. tritici* by using carboxin as selection agent. A 12,530 bp fragment of pCeGFPTub2 (Schuster et al., 2015; digested with *Bsr*GI), 1149 bp *Z. tritici* α-tubulin promoter (amplified with SK-Sep-14 and SK-Sep-47; Table 2), 2073 bp full-length *ztfim1* gene without stop codon (amplified with SK-Sep-240 and SK-Sep-241; Table 2) and 717 bp *egfp* (amplified with SK-Sep-16 and SK-Sep-42; Table 2) were recombined in yeast *S. cerevisiae* to obtain the vector pCeGFPRab5 (Fig. 2C).

The vector pCeGFPRab5 contains *egfp* fused to the full-length *ztrab5* under the control of constitutive *zttub2* promoter and terminator sequences for targeted integration into the *sdi1* locus of *Z. tritici* by using carboxin as selection agent. A 14,907 bp fragment of pCeGFPTub2 (see Schuster et al., 2015; digested with *Xho*I) and 812 bp full-length *ztrab5* gene (amplified with SK-Sep-61 and SK-Sep-62; Table 2) were recombined in yeast *S. cerevisiae* to obtain the vector pCeGFPRab5 (Fig. 2D).

Plasmid pHeGFPRab5 contains *egfp* fused to the full-length *ztrab5* under the control of constitutive *zttub2* promoter and terminator sequences for ectopic random integration by using hygromycin B as selection agent. A 14,343 bp fragment of pCeGFPRab5 (Fig. 2E; digested with *Bam*HI and *Bgl*II) and 1510 hygromycin resistance cassette (amplified with SK-Sep-128 and SK-Sep-129; Table 2) were recombined in yeast *S. cerevisiae* to obtain the vector pHeGFPRab5 (Fig. 2B). Note that this vector was derived from carboxin resistance conferring vector pCeGFPRab5 and as such it contains part of the succinate dehydrogenase gene, carrying the mutation H267L and succinate dehydrogenase terminator. However, these fragments are of no significance.

The vector pCeGFPRab7 contains *egfp* fused to the full-length *ztrab7* under the control of constitutive *zttub2* promoter and terminator sequences for targeted integration into the *sdi1* locus of *Z. tritici* by using carboxin as selection agent. A 14,907 bp fragment of pCeGFPTub2 (see Schuster et al., 2015; digested with *Xho*I) and 815 bp full-length *ztrab7* gene (amplified with SK-Sep-63 and SK-Sep-64; Table 2) were recombined in yeast *S. cerevisiae* to obtain the vector pCeGFPRab7 (Fig. 2D).

The vector pHeGFPRab7 contains *egfp* fused to the full-length *ztrab7* under the control of constitutive *zttub2* promoter and terminator sequences for ectopic random integration by using hygromycin B as selection agent. A 14,655 bp fragment of pCeGFPRab7 (Fig. 2E; digested with *Bgl*II) and 1510 hygromycin resistance cassette (amplified with SK-Sep-128 and SK-Sep-129; Table 2) were recombined in yeast *S. cerevisiae* to obtain the vector pHeGFPRab7 (Fig. 2B). Note that this vector was derived from carboxin resistance conferring vector pCeGFPRab7 and as such it contains part of the succinate dehydrogenase gene, carrying the mutation H267L and succinate dehydrogenase terminator. However, these fragments are of no significance. Further details on vector construction and yeast recombination-based cloning is provided in Kilaru and Steinberg (2015).

### 2.4 *Z. tritici* transformation

The vectors pHFim1eGFP, pHeGFPRab5 and pHeGFPRab7 were transformed into *A. tumefaciens* strain EHA105 by heat shock method (Holsters et al., 1978) and *A. tumefaciens* mediated transformation of *Z. tritici* was performed as described previously by (Zwiers and De Waard, 2001) with the slight modifications. Further details on this method are provided in Kilaru et al. (2015).

### 2.5 Epi-fluorescence microscopy

Fluorescence microscopy was performed as previously described (Schuster et al., 2011a). Fungal cells were grown in YG media either at 18 °C with 200 rpm (for yeast-like cells) or at 24 °C with 100 rpm (for hyphal cells) for ∼24 h. After placing onto a 2% agar cushion, cells were observed using a motorized inverted microscope (IX81; Olympus, Hamburg, Germany), equipped with a PlanApo 100×/1.45 Oil TIRF (Olympus, Hamburg, Germany). Fluorescent tags and dyes were exited using a VS-LMS4 Laser Merge System with solid-state lasers (488 nm/50 mW or 75 mW and 561 nm/50 mW or 75 mW; Visitron Systems, Puchheim, Germany). CMAC (Molecular Probes/Invitrogen, Paisley, UK) was visualized using a standard mercury burner. Z stacks were generated by using a objective piezo (Piezosystem jena GmbH, Jena, Germany). Synchronized observation of red and green fluorescence was performed using an dual imager (Dual-View 2 Multichannel Imaging System; Photometrics, Tucson, USA) equipped with a dual-line beam splitter (z491/561; Chroma Technology Corp., Bellows Falls, USA) with an emission beam splitter (565 DCXR; Chroma Technology Corp., Bellows Falls, USA), an ET-Band pass 525/50 (Chroma Technology Corp., Bellows Falls, USA), and a single band pass filter (BrightLine HC 617/73; Semrock, New York, USA). Images were captured using a CoolSNAP HQ2 camera (Photometrics/Roper Scientific, Tucson, USA) and kymographs were generated using MetaMorph (Molecular Devices, Wokingham, UK). All parts of the system were under the control of the software package MetaMorph (Molecular Devices, Wokingham, UK).

Actin patches were disrupted by incubating the cells in YG media containing 10 μM Latrunculin A (Molecular Probes/Invitrogen, Paisley, UK) for 30 min at 18 °C with 200 rpm. Treated cells were placed onto a 2% agar cushion containing 10 μM Latrunculin A, followed by microscopic observation, using an exposure time of 150 ms and a 488 nm laser at 75% output power.

To co-localize EE with the endocytic marker dye FM4-64 (Molecular Probes/Invitrogen, Paisley, UK), cells were incubated in YG media containing 1 μM FM4-64 for 15 min 18 °C with 200 rpm. The cells were washed by centrifugation for 5 min at 5000 rpm and suspended in fresh YG media. Cells were placed onto a 2% agar cushion and directly observed using the dual-line beam splitter. Images series of 100 planes were acquired, using the 488 nm laser at 100% output power and an exposure time of 150. To examine the effect of the absence of MT on EE motility, cells were incubated in YG media containing 300 μM benomyl (Sigma–Aldrich Chemie Gmbh, Munich, Germany) for 45 min at 18 °C with 200 rpm. Treated cells were observed on 2% agar cushion containing 300 μM benomyl.

Late endosomes and vacuoles labelled with eGFP-Rab7 were counterstained in YG media containing 100 μM vacuolar marker CellTracker Blue CMCA (Molecular Probes/Invitrogen, Paisley, UK) for 15 min at 18 °C with 200 rpm The cells were washed by centrifugation for 5 min at 5000 rpm and re-suspended in fresh YG media followed by taking single images in the DAPI and GFP channel using the mercury burner and an exposure time of 20 ms and 100 ms respectively. Movie of 100 planes in the GFP channel with the 488 nm laser at 100% and an exposure time of 150 ms were taken to visualize late endosome motility.

## 3 Results and discussion

### 3.1 Identification of ZtFim1, ZtRab5 and ZtRab7

In this study, we set out to establish fluorescent marker proteins for the endocytic pathway in *Z. tritici*. We choose the actin-binding protein fimbrin (Fig. 1A; Fim1) and the small GTPases Rab5 and Rab7. The latter are located to early endosomes, late endosomes and vacuoles (Fig. 1A). We identified homologues in *Z. tritici* by screening the published genome sequence (Goodwin et al., 2011; see materials and methods), using the *U. maydis* fimbrin, Fim1 (Castillo-Lluva et al., 2007), and the endocytic Rab-GTPases Rab5a and Rab7 (Fuchs and Steinberg, 2005). This revealed the putative ZtFim1 (protein ID 72868; NCBI accession number: XP_003851390), and the putative GTPases ZtRab5 (protein ID: 66333; NCBI accession number: XP_003857299), and ZtRab7 (protein ID 99493; NCBI accession number: XP_003854495). All predicted *Z. tritici* proteins showed high sequence similarity with their published orthologues in other fungi (Fig. 1B, Table 1) and shared similar domain structures (Table 1). The translational start and the stop of each open reading frame were confirmed by comparison with homologous proteins.

### 3.2 Vectors for random ectopic integration of Fim1-GFP, eGFP-Rab5 and eGFP-Rab7 fusion constructs

In order to visualize actin patches and early, late and recycling endosomes in *Z. tritici*, we constructed the vectors pHFim1eGFP, pHeGFPRab5 and pHeGFPRab7 (Fig. 2A and B). These vectors contain the enhanced green-fluorescent protein (eGFP) fused to ZtFim1, ZtRab5 and ZtRab7. Integration of these vectors into the genome allows the expression of Fim1-eGFP, eGFP-Rab5 and eGFP-Rab7 fusion proteins under the control of constitutive *Z. tritici* α-tubulin promoter (Fig. 2A and B; P*tub2*). The vectors were built on the *Agrobacterium* binary vector pCAMBIA0380 (CAMBIA, Canberra, Australia), which enables *A. tumefaciens*-based transformation into *Z. tritici*, based on the 25 bp imperfect directional repeat sequences of the T-DNA borders (right and left border, RB and LB; Fig. 2A and B). The vectors also carry a kanamycin resistance gene and origins of replication for amplification in *E. coli* and *A. tumefaciens*. We designed these vectors for random ectopic integration into the genome of *Z. tritici*, using hygromycin B as selection agent. These vectors can also be used in combination with other resistance cassette, such as *nptII* (neomycin phosphotransferase; G418-resistant) or *bar* (phosphinothricin acetyltransferase; Basta-resistant) or *sdi1^R^* (mutated allele of succinate dehydrogenase, H267; carboxin-resistant; see Kilaru and Steinberg, 2015 for more details). In addition, all four vectors comprise a “yeast recombination cassette”, consisting of URA3 and 2μ *ori*, which enables yeast recombination-based cloning (for more details see Kilaru and Steinberg, 2015). It needs to be noted that all the above four vectors contain the *sdi1* downstream sequence (*sdi1* left flank and terminator, Fig. 2A and B). This sequence is a remnant of the cloning procedure and has no functional significance.

### 3.3 Visualization of fluorescently-labelled endocytic compartments in *Z. tritici*

In order to visualize actin patches, early endosomes, late endosomes and vacuoles, the vectors pHFim1eGFP, pHeGFPRab5 and pHeGFPRab7 were transformed into *Z. tritici* strain IPO323 (Kema and van Silfhout, 1997) using *A. tumefaciens*-mediated transformation (Zwiers and De Waard, 2001). Transformants were visualized microscopically for the presence of green fluorescence, and positive strains were named IPO323_HFim1eGFP, IPO323_HeGFPRab5 and IPO323_HeGFPRab7 respectively.

**Fig. 3:**
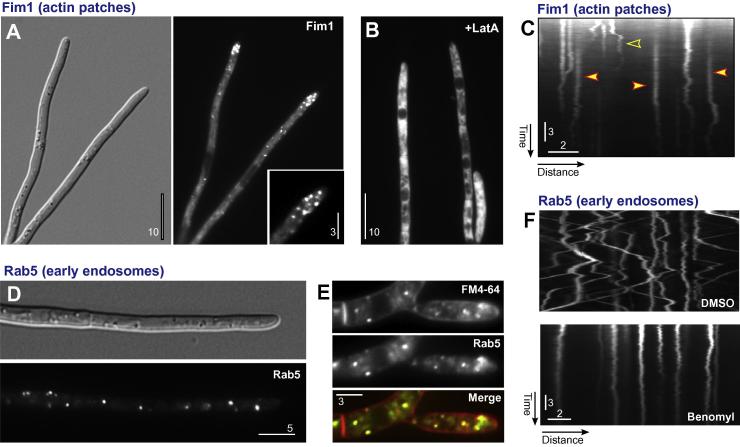
Compartments of the early endocytic pathway in *Z. tritici*. (A) Hyphal cells expressing Fim1-eGFP, after ectopic integration of pHFim1eGFP. The actin-binding protein concentrates in patches at the growth region (see inset). This localization is typical for actin patches in fungi. Bars represent 10 μm and 3 μm. (B) Fim1-eGFP in the presence of the actin-disrupting drug Latrunculin A (incubated at 10 μM for 30 min). Actin-patches disappeared and the fluorescent fusion protein locates in the cytoplasm. Bar represents 10 μm. (C) Kymograph showing motility of Fim1-eGFP signals in *Z. tritici*. Signals remain stationary, but start random lateral movement before they disappear (open arrowhead). Note that several Fim1-eGFP signals appear during the course of observation (filled arrowheads). This motility behavior is consistent with that of fungal actin patches. Bars represent 3 s and 2 μm. (D) Localization of the early endosome marker protein eGFP-Rab5, expressed after ectopic integration of pHeGFPRab5, in a hyphal cell of *Z. tritici*. Bar represents 5 μm. (E) Co-visualization of eGFP-Rab5 (green in overlay) and the endocytic marker dye FM4-64 (red in overlay) in *Z. tritici*. The dye is concentrated in the plasma membrane, from where it is taken up into early endosomes. These organelles co-localize with eGFP-Rab5 (yellow) in overlay. This confirms that the marker labels an early endocytic compartment. Bar represents 3 μm. (F) Kymograph showing motility of eGFP-Rab5-labelled early endosomes. Moving signals are represented by diagonal lines, whereas stationary signals are provide vertical lines. Note that treatment with the microtubule-inhibitor benomyl (300 μM, 45 min) abolishes motility. This is consistent with a microtubule-based transport of early endosomes, reported in other fungal systems. Bars represent 3 s and 2 μm.

We next analyzed the localization of all markers by fluorescent microscopy. Fimbrin localizes to actin patches at the growing hyphal tip of filamentous ascomycetes *Aspergillus nidulans* (Araujo-Bazán et al., 2008; Upadhyay and Shaw, 2008) and *Neurospora crassa* (Delgado-Alvarez et al., 2010) and the growth region of the basidiomycete *U. maydis* (Castillo-Lluva et al., 2007). Consistent with these reports, ZtFim1-eGFP localize in patchy signals in the apical region of hyphae (Fig. 3A). This localization was abolished when F-actin was disrupted with the drug Latrunculin A (Fig. 3B; Spector et al., 1983), which was shown to disassemble actin patches in fungi (Berepiki et al., 2010). Fimbrin is an F-actin-binding protein (Bretscher, 1981) and the dependency of the apical localization of ZtFim1-eGFP on F-actin confirms the correct localization of the marker in actin patches. Furthermore, live cell imaging of ZtFim1-eGFP demonstrated that the fluorescent patches are dynamic (Fig. 3C), with individual signals disappearing (Fig. 3C, open arrowhead) and appearing (Fig. 3C, closed arrowheads) during the course of observation. This behavior is reminiscent of actin patches (Berepiki et al., 2010; Delgado-Alvarez et al., 2010; Kaksonen et al., 2003) overview in (Berepiki et al., 2011; Kovar et al., 2011), again arguing that ZtFim1-eGFP is a suitable marker for visualizing the plasma membrane sites of endocytic uptake.

The small GTPase Rab5 is located on rapidly moving fungal early endosomes (Fuchs and Steinberg, 2005; Abenza et al., 2009; Seidel et al., 2013). In hyphal cells of *Z. tritici*, the putative marker protein eGFP-ZtRab5 concentrates in cytoplasmic spots (Fig. 3D). These were evenly-distributed and moved bidirectionally at 2.18 ± 0.51 μm/s (*n* = 31; anterograde) and 2.36 ± 0.50 μm/s (*n* = 29; retrograde). This velocities are not significantly different from those measured for early endosome motility in *U. maydis* (*P* = 0.6589, Student *t*-test; Schuster et al., 2011b), suggesting that the eGFP-ZtRab5 signals are, indeed, early endosomes. We tested this further by labelling the endocytic pathway of IPO323_HeGFPRab5 cells with the dye FM4-64. This marker is a useful tool to visualize the endocytic pathway in fungi (Fischer-Parton et al., 2000). After short exposure to the cells, the dye inserts into the plasma membrane and, after internalization, passes through the early endosomes (Wedlich-Söldner et al., 2000). We performed similar experiments using IPO323_HeGFPRab5 cells. Indeed, FM4-64 co-localizes with the motile eGFP-ZtRab5-positive organelles (Fig. 3E). Finally, we tested if the motility of these putative early endosomes depends on microtubules, which was previously shown for *U. maydis* (Lenz et al., 2006; Wedlich-Söldner et al., 2000), *A. nidulans* (Abenza et al., 2009) and *N. crassa* (Seidel et al., 2013). Indeed, motility of the eGFP-ZtRab5 labelled structures was abolished when microtubules were depolymerised with the anti-fungal drug benomyl (Fig. 3F). Thus, eGFP-ZtRab5-marked organelles show early endosome specific features, including bi-directional motility at ∼2 μm/s, co-localization with FM4-64 and a dependency of their motility on microtubules. We conclude that eGFP-ZtRab5 is a suitable marker for this endocytic compartment in *Z. tritici*.

**Fig. 4:**
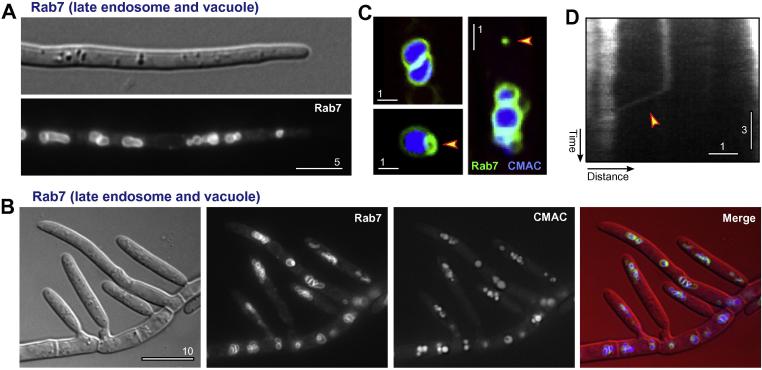
Compartments of the late endocytic in *Z. tritici*. (A) Localization of the late endosome marker protein eGFP-Rab7, expressed after ectopic integration of pHeGFPRab5, in a hyphal cell of *Z. tritici*. Bar represents 5 μm. (B) Co-localization of the late endocytic marker eGFP-Rab7 (green in overlay) and CellTracker Blue CMAC-labelled vacuoles (blue in overlay) in growing macropycnidiospores of *Z. tritici*. Similar to hyphal cells, eGFP-Rab7 localizes predominantly to vacuoles. Bar represents 10 μm. (C) Co-localization of the late endosome marker protein eGFP-Rab7 and the vacuole marker dye CellTracker Blue CMAC. The dye labels the lumen of the Rab7-positive organelles, indicating that they are vacuoles. In addition, unlabelled Rab7-positive signals were found (arrowheads). These were associated with vacuoles (closed arrowhead, lower left) or independent of vacuoles (open arrowhead, right panel). Bars represent 1 μm. (D) Kymograph showing motility of a small eGFP-Rab7-labelled late endosome. Moving signals are represented by diagonal lines, whereas stationary signals are provide vertical lines. Only vacuole-independent Rab7-positive signals showed directed motility. Bars represent 3 s and 1 μm.

Finally, we investigated the localization of the Rab7 homologue ZtRab7. The small GTPase Rab7 is located on late endosomes in mammalian cells (Chavrier et al., 1990) and late endosomes and the fungal vacuole (Abenza et al., 2012; Higuchi et al., 2014). In *Z. tritici*, eGFP-ZtRab7 was mainly localized to the membrane of large compartments that were distributed within the hyphal cell (Fig. 4A) and on budding macropycnidia (Fig. 4B). We co-visualized these compartments with the dye CellTracker blue CMAC (Fig. 4B and C). This showed that the eGFP-ZtRab7-positive structures are vacuoles. This finding is in agreement with the localization of Rab7 in *A. nidulans* (Abenza et al., 2012). In addition, the marker concentrated at vacuole-associated organelles and a few small vesicles (Fig. 4C, arrowheads). These vesicles showed short-range motility at 2.1 ± 0.67 μm/s (Fig. 4D, a kymograph shows motility as a diagonal line; arrowhead). Again, such small Rab7-positive structures were described in *A. nidulans* (Abenza et al., 2012). In summary, eGFP-ZtRab7 shows a localization pattern that is almost identical to that of its homologue in *A. nidulans*.

### 3.4 Vectors for targeted ectopic integration of Fim1-GFP, eGFP-Rab5 and eGFP-Rab7 fusion constructs

As part of the molecular tool set for *Z. tritici*, we constructed another set of vectors, pCFim1eGFP, pCeGFPRab5 and pCeGFPRab7 (Fig. 2Cand D). These plasmids allow targeted integration of the fluorescent marker into the *sdi1* locus, thereby conferring resistance against the fungicide carboxin. Targeted integration into the genomic *sdi1* locus of *Z. tritici* is achieved by a mutated downstream stretch of the *sdi1* sequence, carrying a carboxin resistance conferring point mutation (H267L; Fig. 2C and D, left flank), and a sequence stretch downstream of *sdi1* (Fig. 2C and 2D, right flank of sdi1). Incorporation by homologous recombination mutates the *sdi1* gene and integrates single copies of Fim1-eGFP, eGFP-Rab5, and eGFP-Rab7 constructs into the *sdi1* locus, construct without affecting other *Z. tritici* genes. Again, these vectors enable *A. tumefaciens*-based transformation and yeast recombination-based cloning (see Kilaru and Steinberg, 2015). The vectors are useful for integration into strains that already contain other dominant selectable marker.

## 4 Conclusion

In fungi, endocytosis is an important process that supports hyphal growth (Penalva, 2010; Steinberg, 2014). Invasion of wheat by *Z. tritici* is based on hyphal growth (Mehrabi et al., 2006; Motteram et al., 2011; overview in Steinberg, 2015). Therefore, proteins of the endocytic pathway provide putative targets for novel anti-fungals. Here, we establish fluorescent markers that label all endocytic compartments. We confirm their identity and specificity of their localization by bioinformatics, pharmaceutical experiments, live cell imaging of their dynamics and co-staining with established cellular dyes. Moreover, we show that the cellular localization of all markers corresponds to published data in other filamentous ascomycete fungi. Thus, we conclude that this set of fluorescent proteins is suitable to visualize the organization and dynamic behavior of endocytic compartments in *Z. tritici*. Using these molecular tools will help phenotypic analysis of mutants, but also will support mode-of-action studies of novel fungicides.

## Figures and Tables

**Table 1:** Bioinformatics of putative *Z. tritici* endocytic marker proteins.

	Length^a^		Domains^b^		Identity^c^ (%)	Reference^d^
Fim1	*Z. tritici*	*U. maydis*	*Z. tritici*	*U. maydis*	61.7	Castillo-Lluva et al. (2007)
	658	615	Calponin homology (1.2e−14)	Calponin homology (9.6e−17)		
			Calponin homology (4e−16)	Calponin homology (5e−20)		
			Calponin homology (5.9e−16)	Calponin homology (2.2e−14)		
			Calponin homology (7.7e−09)	Calponin homology (2.6e−11)		

Rab5	*Z. tritici*	*U. maydis*	*Z. tritici*	*U. maydis*	54.0	Fuchs and Steinberg (2005)
	252	280	Ras (7.8e−57)	Ras (4e−54)		

Rab7	*Z. tritici*	*U. maydis*	*Z. tritici*	*U. maydis*	78	Fuchs and Steinberg (2005)
	205	205	Ras (5.4e−56)	Ras (1.4e−56)		

a Given in amino acids.

b Determined in PFAM (http://pfam.xfam.org/search/sequence) with error probability in brackets.

c Determined in EMBOSS Needle (http://www.ebi.ac.uk/Tools/psa/emboss_needle/).

d Reference for comparison.

**Table 2:** Primers used in this study.

Primer name	Direction	Sequence (5′ to 3′)^a^
SK-Sep-14	Sense	*CATTTGCGGCTGTCTCGAAATCGACGGAAG*GCAGTCGACGCCAGATGATGG
SK-Sep-16	Sense	ATGGTGAGCAAGGGCGAGGAG
SK-Sep-42	Antisense	*CCACAAGATCCTGTCCTCGTCCGTCGTCGC*TTACTTGTACAGCTCGTCCATGC
SK-Sep-47	Antisense	GGCGATGGTGGTATGCGGATG
SK-Sep-61	Sense	*ATCACTCTCGGCATGGACGAGCTGTACAAG*ATGGCCGACGCCTCAGCTCCA
SK-Sep-62	Antisense	*CCACAAGATCCTGTCCTCGTCCGTCGTCGC*TCAACAAGCACATCCCTCCTTCG
SK-Sep-63	Sense	ATCACTCTCGGCATGGACGAGCTGTACAAGATGTCATCCAGAAAGAAGATCCTTT
SK-Sep-64	Antisense	*CCACAAGATCCTGTCCTCGTCCGTCGTCGC*CTAGCACGAGCAGCCTTGCTC
SK-Sep-128	Sense	*CTCTCATAAGAGCTTGGCTGTCGACTCCTC*GAATTCGAGCTCGGTACCCAACT
SK-Sep-129	Antisense	*CTTTTCTCTTAGGTTTACCCGCGTTGAAGT*GCGTTAACACTAGTCAGATCTACC
SK-Sep-240	Sense	*CATCACTCACATCCGCATACCACCATCGCC*ATGAACGTGCTCAAGCTGCAGAAG
SK-Sep-241	Antisense	*GGTGAACAGCTCCTCGCCCTTGCTCACCAT*ACCCATCTTCTCCGACGCCGCC

a *Italics* indicate part of the primer that is complementary with another DNA fragment, to be ligated by homologous recombination in *S. cerevisiae*.
